# Successful Primary Repair of a Traumatic Distal Biceps Tendon Rupture With the Arthrex Biceps Button System and Platelet-Rich Plasma Therapy: A Case Report and Surgical Outcome

**DOI:** 10.7759/cureus.87131

**Published:** 2025-07-01

**Authors:** Connor J O'Leary, Pasquale Gencarelli, James M Lee, Rahul Mittal

**Affiliations:** 1 Medicine, Rutgers Robert Wood Johnson Medical School, New Brunswick, USA; 2 Orthopaedic Surgery, Orange Orthopaedic Associates, West Orange, USA; 3 Health Informatics, Rutgers University, Newark, USA

**Keywords:** arthrex button system, distal biceps rupture, distal biceps tendon repair, intraoperative platelet-rich plasma, water skiing

## Abstract

Traumatic rupture of the distal biceps tendon is a relatively rare injury often requiring surgical reconstruction. The typical surgical repair for a traumatic distal biceps tendon rupture is a single anterior incision with cortical button fixation, followed by early mobilization, with most patients returning to full activity or sport at approximately six months postoperatively. We present a case of a 48-year-old male patient who experienced a traumatic distal biceps rupture following a water skiing accident. The patient underwent surgical repair using the Arthrex button system with the application of platelet-rich plasma. Postoperative follow-up showed significant improvement in pain, range of motion, and strength, with the patient returning to normal activities within three months. This case highlights the successful management of a distal biceps rupture and the potential role of platelet-rich plasma in enhancing postoperative recovery.

## Introduction

Distal biceps tendon (DBT) ruptures are relatively uncommon injuries that typically occur in middle-aged males during forceful eccentric contraction of the biceps muscle against a flexed elbow such as during weightlifting or other sporting activities [1]. DBT ruptures typically affect the dominant arm, and individuals involved in athletics or high-load resistance training are particularly susceptible [1,2]. The incidence of DBT rupture has been reported between 1.2 and 5.4 per 100,000 people, and reports of DBT ruptures may be increasing due to a combination of factors, including an aging male population engaging in prolonged physical activity and advancements in clinical and diagnostic imaging [1,3-5]. While a DBT injury is typically precipitated by a forceful contraction event, risk factors, such as tobacco use, increased body mass, use of anabolic steroids, and pre-existing tendinosis, have been linked to an increased risk of DBT injury [1,3,6,7]. DBT ruptures can result in significant function impairment, loss of supination, flexion strength, and decreased resistance to fatigue [8].

Non-surgical treatment of DBT rupture is reserved for those who have low functional demand or are medically unfit to proceed with surgery [9]. It has been shown that healthy, active patients with complete DBT tears benefit from early surgical intervention, yielding improved strength and functional outcomes [10,11]. A variety of surgical plans may be considered for a DBT rupture repair, including anterior, posterior, dual-incision, or endoscopic approaches [12,13]. Less severe cases of acute DBT ruptures may be repaired primarily, while more complex acute and chronic cases may require a graft during surgical repair [5]. There are a number of published cases of DBT rupture repair; however, the level of evidence in most of these reports is regarded as weak [13]. Additionally, as orthopedic technology and techniques have advanced, there are new permutations of approaches for DBT rupture repair. Newer techniques, such as using platelet-rich plasma (PRP), which is an extract derived from the patient’s own blood that is rich in growth factors that facilitate tissue healing, have gained recognition in the conservative management of DBT injuries [14,15]. However, the lack of consensus on the surgical approach for DBT repair and the evolving toolbox of orthopedic approaches calls for more literature to be published on DBT rupture repairs. The purpose of this report is to describe a DBT rupture repair using an Arthrex BicepsButton™ Implant System (Arthrex GmbH, Munich, Germany) with the application of intraoperative PRP.

## Case presentation

We present the case of a 48-year-old right-hand-dominant male with a traumatic rupture of his right DBT sustained while water skiing. The patient reported feeling a pop in his right elbow while water skiing with his arm outstretched. Subsequently, he experienced immediate pain, swelling, and functional impairment. Clinical examination revealed mild swelling in the medial aspect of the elbow, an abnormal appearance of the right biceps, and weakness in supination, pronation, and flexion, consistent with a DBT rupture. The initial evaluation included X-rays, which ruled out fractures or dislocations. An MRI confirmed the diagnosis, showing 4 centimeters of tendon retraction. Given the patient's high-demand lifestyle and desire for restoration of strength, surgical intervention was recommended.

**Figure 1:**
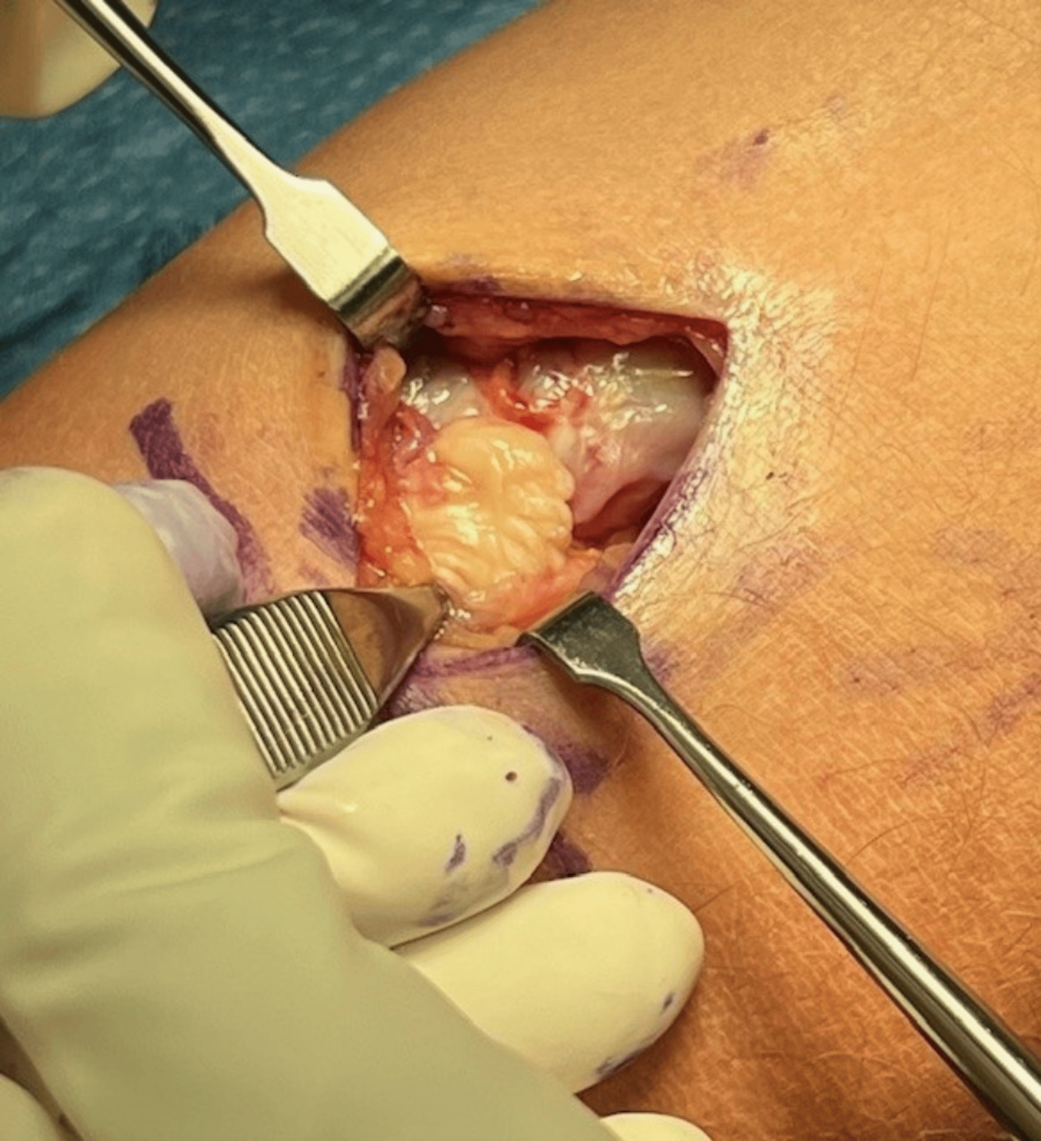
Complete DBT rupture with 4 cm retraction DBT: distal biceps tendon

**Figure 2:**
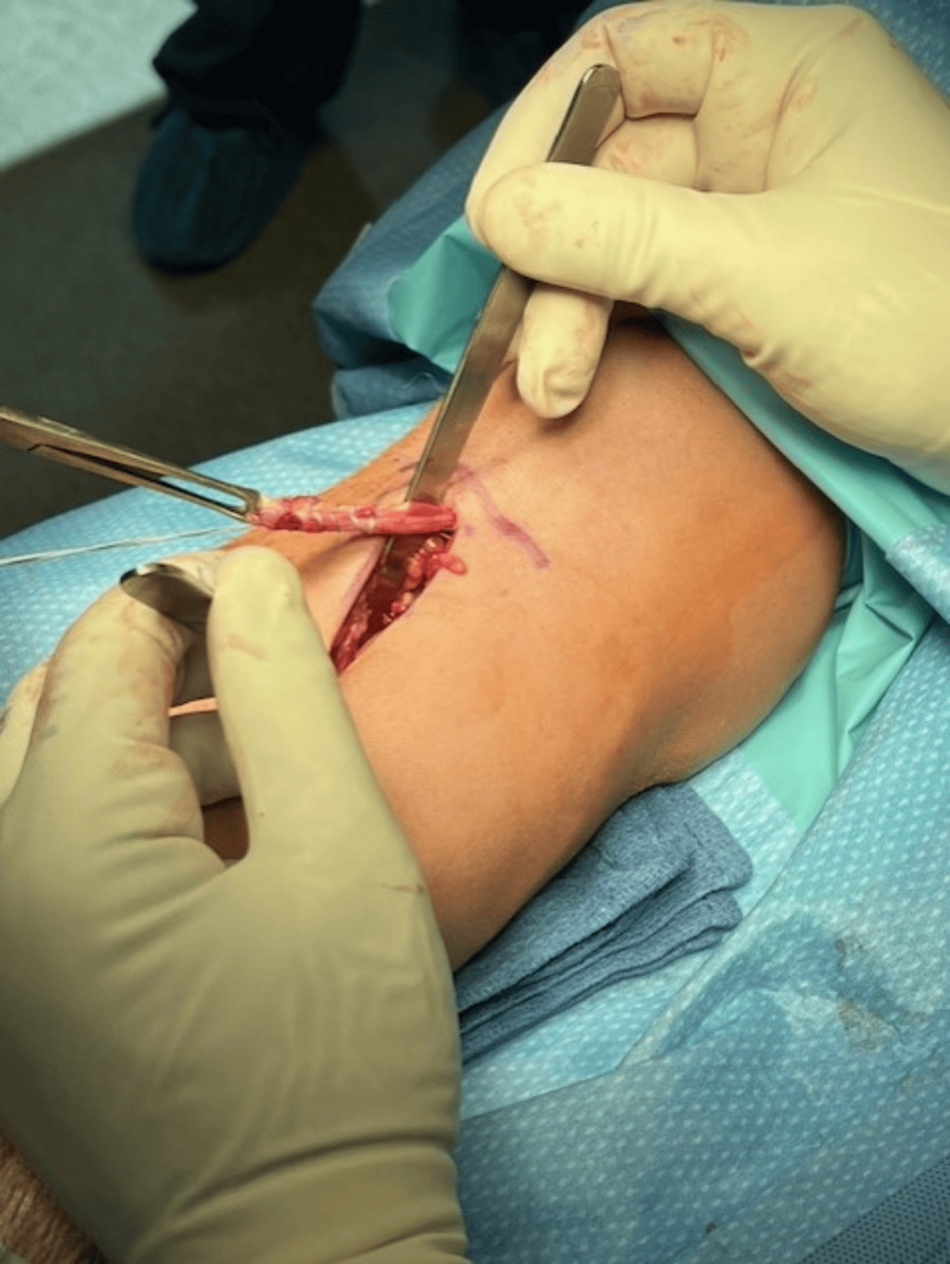
Distal biceps tendon isolation The DBT is isolated through a longitudinal incision distal to the flexion crease and prepared with a whipstitch. DBT: distal biceps tendon

**Figure 3:**
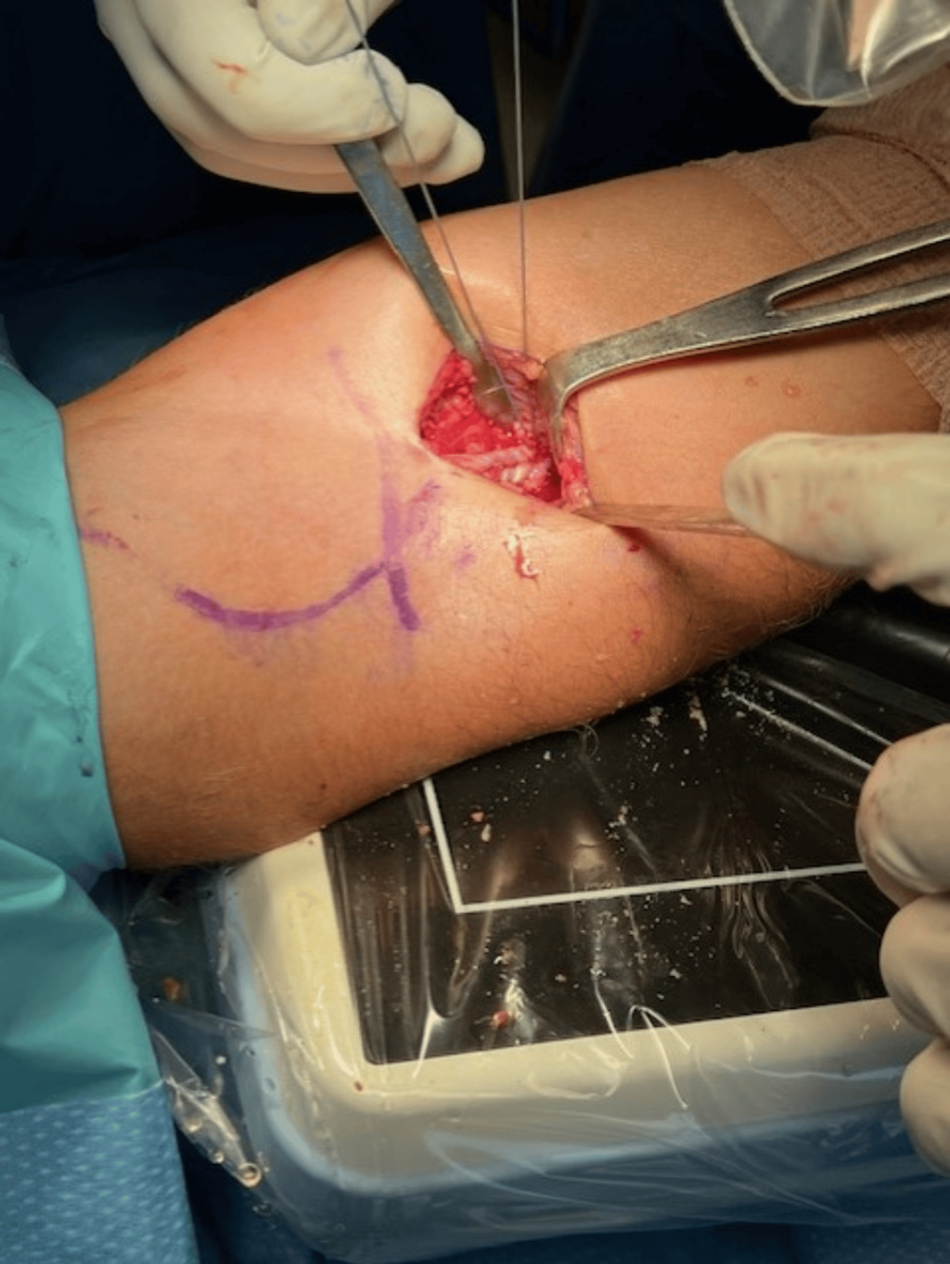
DBT anchored with the Athrex button system DBT: distal biceps tendon

**Figure 4:**
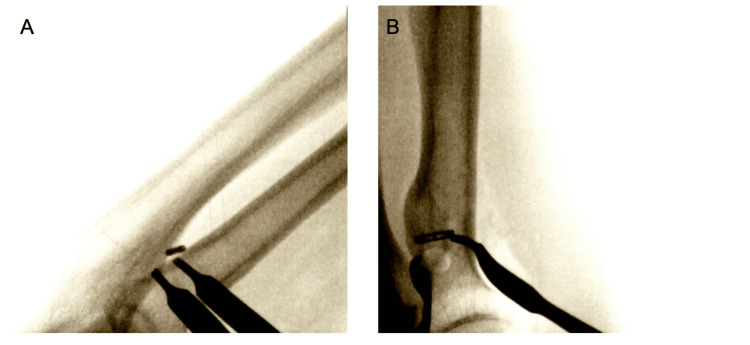
Intraoperative fluoroscopic radiographs demonstrating alignment and confirmation of placement A. Anterior-Posterior; B. Lateral

Two and a half weeks later, the patient underwent primary repair of the distal biceps tendon using the Arthrex BicepsButton™ Implant System, supplemented with the application of PRP. The procedure was performed using a tourniquet under general anesthesia, with the patient in a supine position. A standard longitudinal incision was made distal to the flexion crease, and dissection was carried down to the biceps tendon (Figure 1). Care was taken to protect the lateral antebrachial cutaneous nerve throughout the procedure. After identification of the tendon, any scar tissue was debrided, and the biceps tendon was dissected out. A whipstitch was then placed in the distal biceps and measured (Figure 2). The Arthrex button system was then used for tendon reattachment (Figure 3). With open visualization and fluoroscopic guidance, the bicipital tuberosity was localized, and bicortical drilling was performed. The button was then advanced bicortically and flipped into place. The tendon was advanced into its anatomical location using a tension slide technique, and positioning of the button system was confirmed with fluoroscopy (Figure 4). Additional sutures were placed through the tendon substance to reinforce the repair, and PRP was injected into the repair site to promote healing. The wound was closed in layers, and a well-padded posterior splint was applied to maintain elbow flexion at 90 degrees. Postoperative neurologic examination revealed intact thumb and wrist extension with brisk capillary refill of all digits.

Postoperatively, the patient’s posterior splint was removed at the one-week follow-up visit and replaced with a hinged elbow brace for three weeks. Physical therapy was started after the four-week follow-up visit. The patient reported progressively improving pain, endorsing a 2/10 pain level at the one-week follow-up and a 0/10 pain level at the eight-week follow-up appointment. Functionally, the patient had a 120-degree range of motion in 3 degrees of extension and flexion at the 8-week follow-up visit, while continuing physical therapy. At three months postoperative, the patient reported major progress. Clinical examination revealed a healed incision; full grip strength; full pronation, supination, extension, and flexion; and 5/5 strength in all planes of motion. The patient reported working with physical therapy to be transferred to the gym and was cleared for all activities.

## Discussion

A systematic review by Amarasoryia et al. (2020) determined that after a DBT repair, the overall complication rate was 25%, the major complication rate was 4.6%, and the minor complication rate was 20.4% [16]. With a growing list of fixation systems and adjunct therapies, such as PRP, optimal surgical management of orthopedic conditions like complete DBT ruptures must be continually re-evaluated. While some biomechanical studies have shown that cortical button systems might handle the maximum peak load, suture anchor and interosseous screw techniques showed the least displacement, demonstrating an argument for multiple DBT rupture repair approaches [11]. Currently, there is no consensus regarding the best surgical approach for DBT rupture repair [12,17,18]. Furthermore, the long-term effects of adjunct tissue healing therapies like PRP are unknown. Ultrasound-guided PRP injection therapy has been shown to be safe and effective in patients with distal biceps tendinopathy, but its intraoperative uses have not been explored in the literature [14,15]. While the application of intraoperative PRP has shown promise in orthopedic procedures, such as rotator cuff repairs, its role in DBT rupture repair has not been discussed in the literature [19]. Ultimately, while the specifics of operative approach may vary, surgical repair of DBT rupture remains the standard of care for all patients who can tolerate the procedure. The specific surgical approach taken relies on a combination of an understanding of the distal biceps anatomy, patient assessment, and the surgeon's experience with tendon reattachment techniques [9].

It is crucial to acknowledge the limitations of the existing evidence base, particularly the predominance of low-level evidence in the most cited publications. While our case demonstrated a positive outcome, we don't have long-term data on this patient. Notably, our case provides a single patient experience without a control to serve as a comparison. This highlights the need for further research, including larger prospective cohort studies and randomized controlled trials, to clarify the optimal management strategies for DBT rupture repair.

## Conclusions

This case highlights the successful management of traumatic DBT rupture using a single-incision surgical approach, resulting in favorable outcomes and patient satisfaction. The use of an Arthrex cortical button system and the incorporation of PRP adjunct therapy may offer additional benefits in promoting tissue healing and functional recovery. This case demonstrates a single, successful outcome, but further studies are warranted to evaluate the long-term efficacy and optimal timing of surgical intervention in similar cases.
